# Potential of a Pharmacogenetic-Guided Algorithm to Predict Optimal Warfarin Dosing in a High-Risk Hispanic Patient: Role of a Novel NQO1*2 Polymorphism

**DOI:** 10.1177/2324709616682049

**Published:** 2016-12-01

**Authors:** Dagmar F. Hernandez-Suarez, Karla Claudio-Campos, Javier E. Mirabal-Arroyo, Bianca A. Torres-Hernández, Angel López-Candales, Kyle Melin, Jorge Duconge

**Affiliations:** 1 University of Puerto Rico Medical Sciences Campus, San Juan, PR, USA; 2 University of Puerto Rico Humacao Campus, Humacao, PR, USA

**Keywords:** warfarin, Caribbean Hispanics, pharmacogenetics, NQO1, thrombosis, dosing algorithm

## Abstract

Deep abdominal vein thrombosis is extremely rare among thrombotic events secondary to the use of contraceptives. A case to illustrate the clinical utility of ethno-specific pharmacogenetic testing in warfarin management of a Hispanic patient is reported. A 37-year-old Hispanic Puerto Rican, non-gravid female with past medical history of abnormal uterine bleeding on hormonal contraceptive therapy was evaluated for abdominal pain. Physical exam was remarkable for unspecific diffuse abdominal tenderness, and general initial laboratory results—including coagulation parameters—were unremarkable. A contrast-enhanced computed tomography showed a massive thrombosis of the main portal, splenic, and superior mesenteric veins. On admission the patient was started on oral anticoagulation therapy with warfarin at 5 mg/day and low-molecular-weight heparin. The prediction of an effective warfarin dose of 7.5 mg/day, estimated by using a recently developed pharmacogenetic-guided algorithm for Caribbean Hispanics, coincided with the actual patient’s warfarin dose to reach the international normalized ratio target. We speculate that the slow rise in patient’s international normalized ratio observed on the initiation of warfarin therapy, the resulting high risk for thromboembolic events, and the required warfarin dose of 7.5 mg/day are attributable in some part to the presence of the NQO1*2 (g.559C>T, p.P187S) polymorphism, which seems to be significantly associated with resistance to warfarin in Hispanics. By adding genotyping results of this novel variant, the predictive model can inform clinicians better about the optimal warfarin dose in Caribbean Hispanics. The results highlight the potential for pharmacogenetic testing of warfarin to improve patient care.

## Introduction

Warfarin (Coumadin) is an oral anticoagulant drug that inhibits the vitamin K–dependent clothing factors. To date, it has demonstrated to be highly effective in the treatment and prevention of several thromboembolic disorders, including atrial fibrillation, artificial heart valves, pulmonary embolism, antiphospholipid syndrome, and deep vein thrombosis.^1,2^ Despite the advent of the new direct oral anticoagulants,^3^ warfarin continues to be the mainstay in oral anticoagulation for many patients. According to IMS Health, about 20 million of warfarin prescriptions were dispensed annually in recent years within the Unites States.^4,5^ However, warfarin ranks as one of the top 10 prescription drugs by reports of adverse episodes in outpatients.^6,7^ In spite of its proven clinical utility and the fact that this drug has been on the market for a long time, optimal warfarin dosing continues to be a challenge due to the wide variability among patients, narrow therapeutic index, high potential for drug interactions, high potential for food-based interactions (vitamin K–containing products), and variability in pharmacogenomics.^1,8^

Pharmacogenetic algorithms to predict effective warfarin dose have been developed in several populations worldwide.^1^ Nonetheless, individuals of Hispanic heritage have been largely excluded from derivation cohorts.^9-11^ This seems to exacerbate existing health care disparities due to the omission of certain ethno-specific genetic polymorphisms on relevant pharmacogenes (eg, NQO1*2, CYP4F2*3, CYP2C9*8) of clinical significance among individuals from this minority group.

To our knowledge, there are no reported data regarding the application of pharmacogenetic-guided algorithm in Hispanics with massive abdominal vein thrombosis to determine the optimal warfarin dose. In this report, a recently developed admixture-adjusted pharmacogenetic algorithm for Caribbean Hispanics is applied to a patient carrying a loss of function NQO1*2 (g.559C>T, p.P187S) polymorphism, which seems to be significantly associated with resistance to warfarin in this population.

## Case Presentation

**Figure 1.**
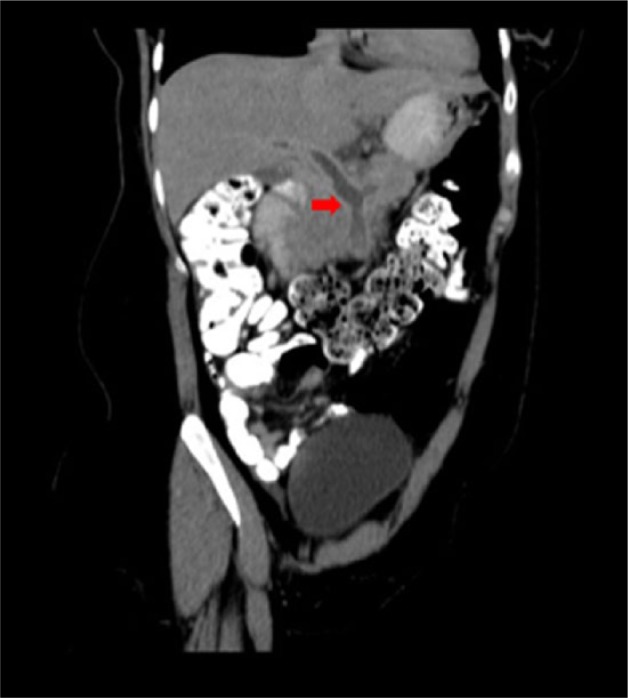
Contrast-enhanced computed tomography of the abdomen and pelvis showing a massive thrombosis of the main portal, splenic, and superior mesenteric veins.

A case is described of a 37-year-old white Hispanic Puerto Rican non-gravid female (147 lbs) with past medical history of abnormal uterine bleeding for 4 months who visited the emergency room due to abdominal pain. The patient mentioned having been evaluated by gynecology service a month ago, and she was started on medroxyprogesterone acetate (Provera, Pfizer, New York, NY), although she just used the medication for 10 days. Moreover, she underwent endometrial curettage procedure and biopsy with no further complications a week before starting with symptoms. She had no history of tobacco, alcohol, or illegal drugs use. On initial evaluation, the patient stated having started with abdominal bloating and upper mild abdominal pain, constant, no radiating, dull, not related with meals or bowel movements, about 2 weeks prior to her visit. She denied nausea, vomiting, fever, anorexia, diarrhea, and constipation. Due to no improvement of symptoms—despite being treated with antacid medications for a suspected gastritis—she went to seek medical attention to the emergency room. Physical examination was just remarkable for unspecific diffuse abdominal tenderness. Additionally, general initial laboratory results were unremarkable with a prothrombin time and partial thromboplastin time of 10.0 and 26.6 seconds, respectively, and international normalized ratio (INR) of 0.9. No electrolyte abnormalities or anemia were observed. As part of an initial imaging workup, a contrast-enhanced abdominal computed tomography was ordered and an acute on chronic massive thrombosis of the main portal, splenic, and superior mesenteric veins was diagnosed (Figure 1). The patient was then admitted under oral anticoagulation therapy with warfarin at 5 mg/day and low-molecular-weight heparin (ie, Lovenox [enoxaparin] injections) 70 mg every 12 hours, the second discontinued at day 7 of therapy. The need to use a short course of low-molecular-weight heparin was due to the initial temporal effect of Coumadin in promoting clot formation as a consequence to the secondary increase of protein C and protein S factors, also dependent of vitamin K. Follow-up laboratories showed normal results in antithrombin III, protein C and protein S activity, antiphospholipid antibodies, factor V Leiden mutation, JAK2 mutation, prothrombin gene mutation, flow cytometry for CD55 and CD59, and direct Coombs test.

On initial evaluation, the patient was approached by our team and agreed to participate as a volunteer in an Institutional Review Board–approved research study aiming to investigate the clinical utility of a recently developed pharmacogenetic-guided algorithm for Caribbean Hispanics.^12^ Consequently, we collected a buccal swap sample and genotype testing was performed after obtaining her written consent. The sample was interrogated for 6 relevant genetic variants on 4 candidate pharmacogenes (ie, *CYP2C9*, *CYP4F2*, *NQO1*, and *VKORC1*) using the corresponding probes from TaqMan SNP assay technology (Applied Biosystems, Foster City, CA). Consequently, she was found to be single carrier of the NQO1*2 (g.559C>T, p.P187S) polymorphism and noncarrier of the the remaining analyzed variants.

**Figure 2.**
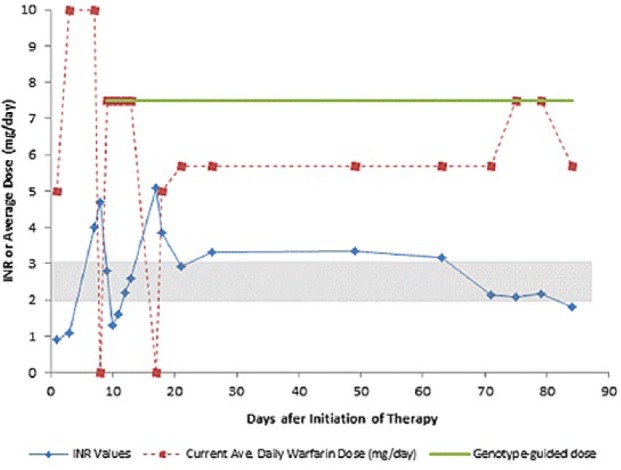
Serial INR values and daily warfarin doses over the follow-up period. The optimal warfarin dose of 7.5 mg/day was predicted (green line) by a pharmacogenetic-guided algorithm. Therapeutic range (2.0-3.0) is represented as a gray shaded area. Routinely prescribed “standard” average daily dose of warfarin (ie, 5 mg/day) is depicted as a dashed line in red.

Based on the diagnosis of deep vein thrombosis, the recommended INR therapeutic range is between 2.0 and 3.0. The patient was initiated on oral warfarin at 5 mg/day following dose recommendations in clinical charts (nomograms) and current American College of Chest Physicians guidelines.^13,14^ The baseline INR value on admission was 0.9 (Table 1). Consecutive INR value of 1.1 was measured on day 3 of therapy and the warfarin dose was increased to 10 mg/day (the highest dose given). Following the pharmacogenetic-driven algorithm recommendations, the patient was prescribed warfarin at the dose of 7.5 mg/day for further prevention of thrombus formation. She was kept on this dose until discharge when she reported 2 continue INR values on therapeutic range. Notably, the dose prescribed at the time of achieving the first target INR was 7.5 mg/day (Figure 2). It is important to mention that during the follow-up period, warfarin dosing was stopped on 2 occasions. First, on day 7 due to an INR value of 4.0 (patient was on 10 mg/day) and on day 17 after a reported INR of 5.09.

**Table 1.** Consecutive International Normalized Ratio (INR) Measurements and Daily Warfarin Dose Over the Entire Follow-up Period (ie, 12 weeks). Therapeutic Range Was Set at 2.0 to 3.0.

No.	Day/Week	Test Date	INR Value	Dosing (mg/day)
1	Baseline	March 26, 2016	0.9^a^	5 mg/day
2	Day 3/week 1	March 28, 2016	1.1^a^	Rise to 10 mg/day
3	Day 7/week 1	April 1, 2016	4.0^b^	10 mg/day and then stop warfarin
4	Day 8/week 1	April 2, 2016	4.7^b^	One day off
5	Day 9/week 2	April 3, 2016	2.8^c^	Resume warfarin at 7.5 mg/day
6	Day 10/week 2	April 4, 2016	1.3^a^	7.5 mg/day
7	Day 11/week 2	April 5, 2016	1.6^a^	7.5 mg/day
8	Day 12/week 2	April 6, 2016	2.2^c^	7.5 mg/day
9	Day 13/week 2	April 7, 2016	2.6^c^	7.5 mg/day and discharged
10	Day 17/week 3	April 11, 2016	5.09^b^	Stop warfarin for 1 day
11	Day 18/week 3	April 12, 2016	3.86^b^	Resume warfarin at 5 mg/day
12	Day 21/week 3	April 15, 2016	2.93^c^	5 mg Monday to Friday; 7.5 mg Saturday and Sunday
13	Day 26/week 4	April 20, 2016	3.30^b^	5 mg Monday to Friday; 7.5 mg Saturday and Sunday
14	Week 7	May 8, 2016	3.35^b^	5 mg Monday to Friday; 7.5 mg Saturday and Sunday
15	Week 9	May 26, 2016	3.17^b^	5 mg Monday to Friday; 7.5 mg Saturday and Sunday
16	Week 11	June 4, 2016	2.15^c^	5 mg Monday to Friday; 7.5 mg Saturday and Sunday
17	Week 11	June 8, 2016	2.07^c^	7.5 mg/day
18	Week 12	June 11, 2016	2.18^c^	7.5 mg/day
19	Week 12	June 16, 2016	1.81^a^	5 mg Monday to Friday; 7.5 mg Saturday and Sunday

a Below therapeutic range.

b Above therapeutic range.

c INR levels on target.

As per protocol, a 3-month follow-up period was undertaken for identifying warfarin-related adverse episodes as well as other relevant on-treatment events in the patient. Warfarin dosing was changed 6 times in this patient during the course of 90 days. There were 19 INR assessments, with 4 tests made before the patient reached first INR on target (Table 1). Thirty-seven percent of these INRs exceeded target range, peaking at 5.09. Last INR measured on week 12 was 1.81, suggesting a mild subtherapeutic level of warfarin dosage, and re-exposing her to a slightly higher risk of thrombosis. Previous reports suggest a 17-fold increase in stroke as INR fell below 2.0, whereas the risk for major and life-threatening bleeding events increases 42% for each unit of INR increment above therapeutic range.^15^ Fortunately, in this case neither strokes nor thromboembolic events or major life-threatening bleeding episodes were reported. Over the follow-up period, the patient reported 2 bruises (one in the calves and the other in the thighs) on weeks 2 to 4, moderate palpitations and dark stools on week 3, chest pain on weeks 3 and 4 and weeks 11 and 12, as well as recurrent fatigue, weakness, anxiety, and discomfort.

## Discussion

Deep abdominal vein thrombosis is extremely rare among thrombotic events secondary to the use of contraceptives.^16^ Patients with this condition are at high risk of mortality due to the resulting extensive intestinal ischemia secondary to the blood flow obstruction. Thus, it represents a medical emergency that needs to be treated immediately and yet the mortality rate remains significant.^17^ Furthermore, the unspecific abdominal symptoms—often referred as the main complaint—and findings on physical examination make difficult the diagnosis, which results in frequent delays in therapy. To date, prompt anticoagulation represents the mainstay therapy. Nevertheless, bowel surgical resection might become the most appropriate choice when associated surgical complications are present. In the present case, we introduced for the first time genetic information to guide the anticoagulation therapy in a Hispanic female with an atypical thrombosis.

### Pharmacogenetic Analysis

An admixture-adjusted pharmacogenetic algorithm that explained more than two thirds of the observed variance in stable warfarin dose within Caribbean Hispanics was recently developed by our team.^12^ Among predictors of warfarin dose variability in this model, the effect on dose of all *CYP2C9* polymorphisms combined was −18%, whereas the effect of both *VKORC1*-AA and *VKORC1*-GA status combined was −13%. CYP4F2*3 18000G>A (V433M, rs2108622) and NQO1*2 c.559C>T (P187S, rs1800566) variants were independently associated with a 17% and 10% increase of the dose per variant allele, respectively, whereas the admixture index decreases the dose by 7%. In the proposed model, the effect (%) on the estimates of the effective dose is calculated per number of variant alleles (ie, *CYP2C9*, *CYP4F2*, *NQO1*, and *VKORC1*), per decades (Age) and per 0.25-unit increase in the dose-adjusted INR response at the third day.

The observed frequency of the NQO1*2 (g.559C>T, p.P187S, rs1800566) “resistant” polymorphism among the cardiovascular patients from the Puerto Rican population used as the “derivation cohort” was 0.10 (95% confidence interval [CI] = 0.07-0.13).^12^ There are 15.7% carriers of the C/T heterozygous genotype for the NQO1*2 SNP (95% CI 0.12-0.21) and 2% were double carriers (T/T) of this variant (95% CI = 0.007-0.05).^12^ The NAD(P)H dehydrogenase, quinone 1 (*NQO1*) gene encodes for a FAD-binding cytoplasmic 2-electron reductase enzyme that catalyzes the reduction of vitamin K to its active hydroquinone form, and it is therefore involved in the vitamin K–dependent prothrombin synthesis.^18,19^ Notably, the combination of CYP4F2*3 and NQO1*2 “resistant” variants, identified in as many as 20.4% and 17.7% of individuals from the Puerto Rican study cohort, respectively, had only been previously described to be associated with higher warfarin dose requirements in another US Hispanic population of Mexican descents.^10^

The prediction of 7.5 mg/day of maintenance warfarin by the developed pharmacogenetic-guided algorithm coincided with the patient’s actual effective warfarin dose to reach the INR therapeutic target. The delayed increase in the INR levels of this patient during the initial phase of therapy—in addition to the life cycle of existing clotting factors—may be, in part, a consequence of the NQO1*2 (g.559C>T, p.P187S) polymorphism found in this subject. Interestingly, this genetic variant seems to be significantly associated in Hispanics with resistance to warfarin.^10,12^

Although American College of Chest Physicians guidelines recommends against routine use of pharmacogenetic testing for guiding doses of warfarin in patients initiating therapy (Grade 1B),^14^ the new and revised 2014 American Heart Association/American Stroke Association guidelines for the primary prevention of stroke recommend that pharmacogenetic dosing of vitamin K antagonists may be considered when therapy is initiated (level of evidence C).^20^ According to the Clinical Pharmacogenomics Implementation Consortium (CPIC) guidelines for warfarin maintenance dosing, the recommendations based on genetic information are rated as level A or strong, and are derived from numerous observational studies and some prospective trials that suggest the ability to more accurately identify stable therapeutic warfarin dose requirements through the use of both individual genotypes and clinically relevant information.^1^ The CPIC guideline also indicates that the use of pharmacogenetic algorithm-based dosing is a better predictive tool and, therefore, is recommended over other methods as long as clinical decision support systems or electronic means are available. This CPIC guideline is currently under revision to update recommendations and incorporate new evidences. Nevertheless, at the moment, there should be some level of consideration for the feasibility of pharmacogenetic testing in the context of warfarin treatment across various health care settings, as widespread pharmacogenetic testing in the outpatient setting may not be feasible or cost-effective.

Most of the currently available pharmacogenetic-driven warfarin dosing algorithms overlook the effect of polymorphisms occurring on pharmacogenetic loci not found to be strongly associated with warfarin dose requirements among White Europeans,^21-25^ as opposed to the common CYP2C9*2, CYP2C9*3, and *VKORC1*-1639G>A variants that are indeed systematically incorporated into the developed models. It is becoming increasingly clear that the utility of existing models is limited in patients with mixed ancestry like Caribbean Hispanics or African Americans. A previously reported clinical trial (COAG, NCT00839657) raised concerns about real benefits from genotyping patients on warfarin to optimal dose predictions.^25^ Nonetheless, we strongly believe the COAG trial failed to account for certain ethno-specific genetic polymorphisms of clinical relevance, which led to significant errors in dosing predictions among individuals of African heritage. Accordingly, it is fairly reasonable to expect the same poor predictability by ignoring admixture and novel pharmacogenetic markers (eg, NQO1*2) of potential clinical interest in patients from underrepresented populations like Caribbean Hispanics.

## Conclusions

The case reported supports our perception about the predictability of our pharmacogenetic model, after adding the genotyping result of this novel variant, in order to better inform clinicians on optimal warfarin dosing in the Hispanic population. Additionally, it highlights the potential for pharmacogenetic testing of warfarin to improve patient care and reduced health care costs. Further studies are warranted to determine whether the applied algorithm in this case could decrease adverse outcomes, hospital stay, and contribute as an important decision support tool on initiation of warfarin therapy.
